# ISAR Imaging of Maneuvering Targets Based on the Modified Discrete Polynomial-Phase Transform

**DOI:** 10.3390/s150922401

**Published:** 2015-09-03

**Authors:** Yong Wang, Ali Cherif Abdelkader, Bin Zhao, Jinxiang Wang

**Affiliations:** Research Institute of Electronic Engineering Technology, Harbin Institute of Technology, Harbin 150001, China; E-mails: Abdelkader123@163.com (A.C.A.); zhaobinhit1988@hit.edu.cn (B.Z.); jxwang@hit.edu.cn (J.W.)

**Keywords:** ISAR, maneuvering target, PPS, DPT

## Abstract

Inverse synthetic aperture radar (ISAR) imaging of a maneuvering target is a challenging task in the field of radar signal processing. The azimuth echo can be characterized as a multi-component polynomial phase signal (PPS) after the translational compensation, and the high quality ISAR images can be obtained by the parameters estimation of it combined with the Range-Instantaneous-Doppler (RID) technique. In this paper, a novel parameters estimation algorithm of the multi-component PPS with order three (cubic phase signal-CPS) based on the modified discrete polynomial-phase transform (MDPT) is proposed, and the corresponding new ISAR imaging algorithm is presented consequently. This algorithm is efficient and accurate to generate a focused ISAR image, and the results of real data demonstrate the effectiveness of it.

## 1. Introduction

High resolution inverse synthetic aperture radar (ISAR) imaging is a critical tool to generate a focused image of a moving target [1,2,3,4,5,6,7,8], and it is widely used in the fields of target recognition, space probing and aircraft traffic control, *etc.* [9,10,11]. In the past three decades, many useful results have been obtained by scholars all around the world [12,13,14,15,16,17]. The high resolution in the range coordinate is achieved by transmitting a large bandwidth coded signal, and the high cross range resolution is achieved by the coherent integration of echoes collected at different viewing angles between the radar and target [18]. The primary step before ISAR imaging is the motion compensation, which includes the envelope alignment and the phase adjustment. The envelope alignment can compensate the translational motion of each scatterer after the range is compressed, and the phase adjustment can eliminate the phase errors between two adjacent echoes. The most commonly used methods for the envelope alignment include the accumulated form of maximum correlation algorithm [19], the global algorithm [20] and the minimum entropy algorithm [21]. The phase adjustment can be implemented efficiently by the constant phase error elimination algorithm [19].

The Range-Doppler (RD) algorithm can be adopted to generate a focused ISAR image with the assumption that the Doppler frequency of each scatterer is constant during the observation time, and the Fourier transform is used in the azimuth processing to generate a two-dimensional ISAR image. The RD algorithm is valid for a target with uniform motion. However, when the target has a non-uniform rotation, the Doppler frequency of each scatterer is time-varying, and the RD algorithm will be ineffective because of the spreading effect of the Fourier transform. To solve this problem, the Range-Instantaneous-Doppler (RID) algorithm has been proposed to improve the ISAR image quality, where the Fourier transform is substituted by the time-frequency representations [22,23].

The first approach for the implementation of the RID algorithm is based on the time frequency distributions with high concentration and reduced cross-terms, such as the instantaneous imaging methods based on the Short Time Fourier Transform (STFT) [24], the Wigner-Ville distribution (WVD) [25] and some kinds of high order time frequency distributions [26,27]. These kinds of algorithms have the advantage of computational efficiency, but they still suffer from the tradeoff between the time frequency concentration and the cross-terms. The second approach is based on the parameters estimation technique, where the echo is characterized as multi-component polynomial phase signal (PPS) after the translational compensation, and the high quality ISAR images can be obtained by the parameters estimation of the received signals. When the maneuverability of a non-cooperative target is not too severe, the echo signals can be approximated as the multi-component linear-frequency-modulated (LFM) signals, and the corresponding ISAR imaging algorithms based on the Hough-Wigner transform [28], the match Fourier transform [29], RELAX dechirping method [30] and CLEAN dechirping method [31] have been proposed. These algorithms are effective for the enhancement of ISAR image quality compared with the classical RD algorithm. For a target with high maneuverability, the high order terms will exist in the azimuth echoes, and the LFM signal model is not precise enough to generate a well focused ISAR image in this case. Then, the cubic phase signal (CPS) model was proposed as an alternation to improve the ISAR image quality. The corresponding ISAR imaging algorithms based on the CPS model include the Product-High-Order-Matched-Phase-Transform (PHMT) [32], the keystone time-chirp rate distribution [7], the chirp rate-quadratic chirp rate distribution [8], the local polynomial Wigner distribution [33] and the Product-Generalized-Cubic-Phase-Function (PGCPF) [34], *etc*. These algorithms are more accurate than the LFM signal model in the ISAR imaging of a maneuvering target, but some of them suffer from the high order of nonlinearity and the computational complexity.

Considering the computational complexity and accuracy, a novel parameters estimation algorithm of multi-component PPS with order three (referred as the CPS model, which is commonly used in ISAR imaging of maneuvering target) based on the modified discrete polynomial-phase transform (MDPT) is proposed in this paper, and the corresponding new ISAR imaging algorithm is presented consequently. The discrete polynomial-phase transform (DPT) was proposed in [35,36] to estimate the parameters of multi-component signals, but the performance of it will be degraded when the amplitudes of different components are close to each other. In this paper, we propose a new technique to overcome this disadvantage of the DPT algorithm by the integration operation of different time lags, and the parameters estimation accuracy can be improved greatly with a little increase of computational complexity. The new method is used in ISAR imaging of a maneuvering target, and the image quality is improved. This paper is organized as follows: in Section 2, the PPS model is constructed for the ISAR imaging of maneuvering target; In Section 3, the parameters estimation algorithm of multi-component CPS model based on the MDPT is proposed; In Section 4, the corresponding novel ISAR imaging algorithm is presented; In Section 5, the experimental results for the real data are given; Section 6 is the conclusion of the paper.

## 2. Signal Model Construction

**Figure 1:**
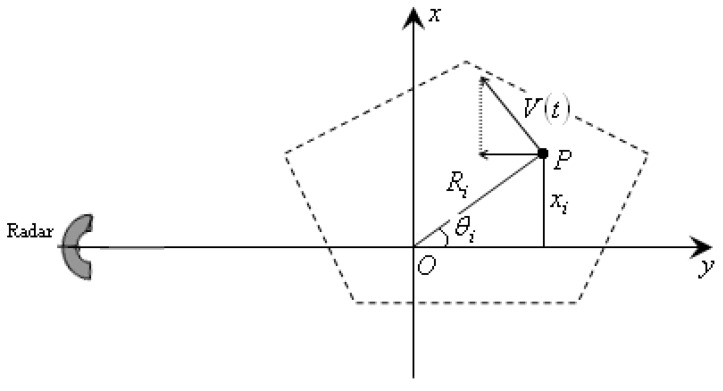
Geometry of radar image of maneuvering target.

It is assumed that the motion compensation has been implemented, and all the scatterers stay in the right range cells. The ISAR imaging geometry can be illustrated in Figure 1. After the motion compensation, a turn-table target model can be obtained with the geometric center O. In the case of maneuvering movement, the angular velocity of the target is time varying, and this will be discussed in this section. The radar is located on the target plane along *Y*-axis. We assume that the target has a non-uniform rotation, and the rotational velocity, acceleration, acceleration rate and higher order terms are $w_{0},w_{1},w_{2},w_{3},...$, respectively. Thus, the radial velocity of a random scatterer P on the target can be computed as Equation (1) as follows:

(1) $$V\left(t\right)=\left(w_{0}+w_{1}t+w_{2}t^{2}+w_{3}t^{3}+\ldots \right)R_{i}$$

where t is the azimuth time, $R_{i}$ is the distance between scatterer P and the geometric center O. The composite velocity V(t) can be decomposed into two parts: one is parallel to the *Y*-axis, which can induce the Doppler frequency of scatterer P with cross-range coordinate $x_{i}$; the other is perpendicular to the *Y*-axis, which has no effect on the Doppler frequency. Hence, the effective velocity can be written as Equation (2) as follows:

(2) $$V_{y}\left(t\right)=V\left(t\right)\sin\theta_{i}=\left(w_{0}+w_{1}t+w_{2}t^{2}+w_{3}t^{3}+\ldots \right)R_{i}\sin\theta_{i}=\left(w_{0}+w_{1}t+w_{2}t^{2}+w_{3}t^{3}+\ldots \right)x_{i}$$

Then, the azimuth echo in a range bin can be written as:

(3) $$s\left(t\right)=\sum_{i=1}^{K}A_{i}\exp\left[j\frac{4\pi }{\lambda }x_{i}\left(w_{0}t+\frac{1}{2}w_{1}t^{2}+\frac{1}{3}w_{2}t^{3}+\ldots \right)\right]$$

where $A_{i}\left(i=1,2,\ldots ,K\right)$ is the amplitude of each scatterer, and K is the number of scatterers in this range bin. It is obvious that the echo signal in a certain range bin has the form of multi-component PPS for the maneuverability of the target, and in this case, the RID algorithm can be used to generate a focused instantaneous ISAR image by the parameters estimation of the PPS.

The parameter estimation of multi-component PPS has been a hot topic in the domain of signal processing, and many algorithms, such as the optimal maximum likelihood algorithm, the product high order ambiguity function algorithm and multilinear function have been proposed recently [37,38,39]. These algorithms are efficient and accurate to estimate the parameters of PPS with arbitrary order. Whereas, the reasonable order of the PPS in ISAR imaging of maneuvering target is three, and this is accurate enough to generate a focused ISAR image with the consideration of computational complexity. In this case, the azimuth echo in a range bin can be approximated as multi-component PPS with order three (CPS model). Hence, we can approximate Equation (3) as (4):

(4) $$s\left(t\right)=\sum_{i=1}^{K}A_{i}\exp\left[j\left(a_{i,1}t+a_{i,2}t^{2}+a_{i,3}t^{3}\right)\right]$$

where the phase coefficients are expressed as Equation (5):

(5) $$\left\{ \begin{array}{l} a_{i,1}=\frac{4\pi }{\lambda }w_{0}x_{i}\\ a_{i,2}=\frac{2\pi }{\lambda }w_{1}x_{i}\\ a_{i,3}=\frac{4\pi }{3\lambda }w_{2}x_{i} \end{array}\right.$$

The high quality ISAR images can be obtained by the parameters estimation of s(t) combined with the RID technique.

## 3. Parameters Estimation of Multi-Component CPS Based on the MDPT

In this section, the parameters estimation of multi-component CPS based on the MDPT is presented.

### 3.1. The Definition of the DPT

The DPT is a tool to analyze the constant amplitude PPS, and it is described in [27]. We assume that s(n) is the discrete form of s(t), τ and M are positive integers. The operators $DP_{1}\left[s\left(n\right),\tau \right]$ and $DP_{2}\left[s\left(n\right),\tau \right]$ are defined as Equation (6) and (7):

(6) $$DP_{1}\left[s\left(n\right),\tau \right]=s\left(n\right)$$

(7) $$DP_{2}\left[s\left(n\right),\tau \right]=s\left(n\right)s^{*}\left(n-\tau \right)$$

where ∗ denotes the conjugate. The higher order operators are defined as Equation (8):

(8) $$DP_{M}\left[s\left(n\right),\tau \right]=DP_{2}\left[DP_{M-1}\left[s\left(n\right),\tau \right],\tau \right]$$

where τ is the delay parameters, M is the order of the operator.

Then, the DPT of order M can be defined as the discrete Fourier transform of $DP_{M}\left[s\left(n\right),\tau \right]$ as Equation (9):

(9) $$DPT_{M}\left[s\left(n\right),\Omega ,\tau \right]=\sum_{n}DP_{M}\left[s\left(n\right),\tau \right]\exp\left(-j\Omega n\Delta \right)$$

where Δ is the sampling interval, and it is assumed that Δ=1 in the following analysis.

### 3.2. Parameters Estimation of Multi-Component CPS Based on the MDPT

Consider a cubic phase signal with the discrete form as Equation (10):

(10) $$s\left(n\right)=A\exp\left[j\left(a_{1}n+a_{2}n^{2}+a_{3}n^{3}\right)\right],0\leq n\leq N-1$$

where A is the amplitude, $a_{1},a_{2},a_{3}$ are the phase coefficients, N is the signal length. The order of the CPS is M=3. Then, we have Equation (11):

(11) $$DP_{3}\left[s\left(n\right),\tau \right]=\exp\left[j\left(\omega_{0}n+\phi_{0}\right)\right],2\tau \leq n\leq N-1$$

where the frequency and initial phase can be expressed as Equation (12) and (13):

(12) $$\omega_{0}=6\tau^{2}a_{3}$$

(13) $$\phi_{0}=2\tau^{2}a_{2}-6\tau^{3}a_{3}$$

Substitute Equation (11) into Equation (9), and we can obtain the estimated value of $a_{3}$ as Equation (14):

(14) $${\hat{a}}_{3}=\frac{1}{6\tau^{2}}\underset{\Omega }{\arg\max}\left\{\left|DPT_{3}\left[s\left(n\right),\Omega ,\tau \right]\right|\right\}$$

Then, the cubic phase signal can be dechirped into a LFM signal with the estimated value of $a_{3}$, and we can proceed to use the DPT algorithm to estimate the other parameters. This will be discussed in detail in the next section.

It is obvious that the DPT is a nonlinear transform, and for a multi-component CPS, the cross-terms will appear. This will affect the parameters estimation accuracy of the individual signal components, especially in the case of the similar amplitudes of different components. The DPT has good performance when one of the signal components is significantly stronger than the others, but the performance of it will be degraded greatly when the amplitudes of different components are close to each other. In this section, we propose a new technique to overcome this disadvantage of DPT algorithm by the integration operation of different time lags, and the parameters estimation accuracy can be improved greatly with a little increase of computational complexity. The modified version of DPT (MDPT) can be written as Equation (15):

(15) $$MDPT_{M}\left[s\left(n\right),\Omega \right]=\sum_{\tau_{m},\tau_{m}\in \Gamma }DPT_{M}\left[s\left(n\right),\Omega ,\tau_{m}\right]$$

where $\Gamma =\left\{\tau_{1},\tau_{2},\ldots ,\tau_{m},\ldots \right\}$ is the sets of delay parameters.

The integration operation can amplify the individual signal components and suppress the cross-terms simultaneously. Therefore, the MDPT is more appropriate for the parameters estimation of multi-component cubic phase signal than the traditional DPT algorithm.

Remark 1: The DPT can be implemented efficiently by the fast Fourier transform (FFT). Hence, it has low computational load compared with some traditional algorithms, such as the maximum-likelihood algorithm, the chirp rate-quadratic chirp rate distribution, the local polynomial Wigner distribution and the PGCPF algorithm, *etc.* These traditional algorithms require a three-dimensional (3D) or a two-dimensional (2D) maximization to estimate the parameters of a cubic phase signal.

### 3.3. Numerical Examples

Some numerical examples are provided here to illustrate the performance of the DPT and MDPT algorithms.

Example 1: Consider a discrete two-component LFM signal with the same amplitudes, which can be written as Equation (16):

(16) $$s\left(n\right)=A_{1}\exp\left[j\left(a_{1,1}n+a_{1,2}n^{2}\right)\right]+A_{2}\exp\left[j\left(a_{2,1}n+a_{2,2}n^{2}\right)\right],0\leq n\leq N-1$$

where N is the length of the signal and it is assumed that N=255. We assume that the sampling rate is one, and the other parameters of s(n) are shown in Table 1.

**Table 1:** Parameters of the two-component LFM signal.

Components (*i*)	*A_i_*	*a_i_*_,1_	*a_i_*_,2_
1	1.5	0.3	2×10^–3^
2	1.5	−0.2	−7×10^–3^

**Figure 2:**
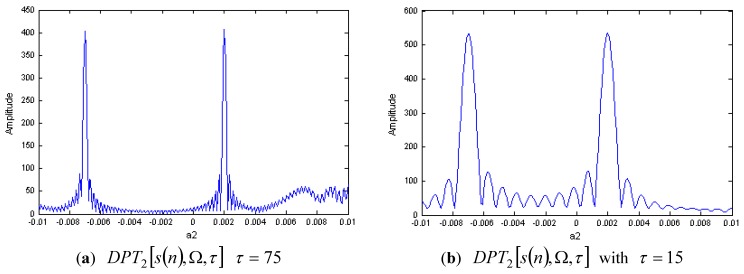
DPT of a two-component LFM signal with different delay parameters.

The DPT of s(n) with M=2 is shown in Figure 2a,b, respectively. In Figure 2a, we select the delay parameter as τ=75, and in Figure 2b, the delay parameter is selected as τ=15. We can see that the second order coefficient $a_{2}$ can be estimated by the peak of $\left|DPT_{2}\left[s\left(n\right),\Omega ,\tau \right]\right|$, and there is no spurious peak in this case.

Example 2: Consider a discrete two-component cubic phase signal with different amplitudes, which can be written as Equation (17):

(17) $$s\left(n\right)=A_{1}\exp\left[j\left(a_{1,1}n+a_{1,2}n^{2}+a_{1,3}n^{3}\right)\right]+A_{2}\exp\left[j\left(a_{2,1}n+a_{2,2}n^{2}+a_{2,3}n^{3}\right)\right],0\leq n\leq N-1$$

The sampling rate is one, and the length of s(n) is N=255. The other parameters of s(n) are shown in Table 2.

**Table 2:** Parameters of the two-component cubic phase signal.

Components (*i*)	*A_i_*	*a_i_*_,1_	*a_i_*_,2_	*a_i_*_,3_
1	1.5	0.3	2×10^–3^	2×10^–5^
2	2.5	0.7	–5×10^–3^	–2×10^–5^

**Figure 3:**
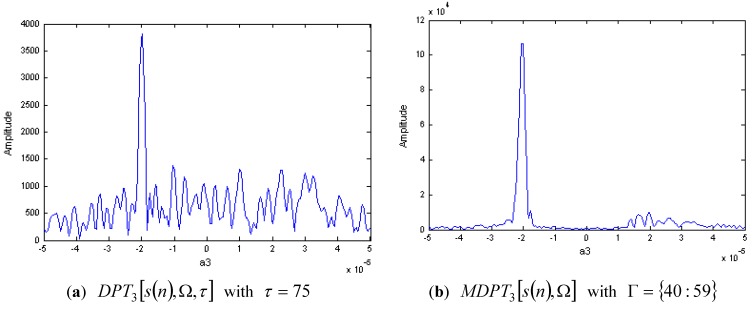
DPT and MDPT of a two-component CPS with different amplitudes.

The DPT of s(n) with M=3 is shown in Figure 3a,b, respectively. In Figure 3a, we select the delay parameter as τ=75, and the third order coefficient $a_{3}$ for the CPS with stronger energy can be estimated by the peak of $\left|DPT_{3}\left[s\left(n\right),\Omega ,\tau \right]\right|$. This demonstrates the effectiveness of DPT algorithm for the signal components with different amplitudes. Figure 3b is the MDPT with Γ={40:59}, we can see that the cross-terms have been reduced greatly, and this is advantageous for the signal detection and parameters estimation.

Example 3: Consider a discrete two-component cubic phase signal with the same amplitudes, as shown in Equation (17), and the parameters are shown in Table 3.

**Table 3:** Parameters of the two-component cubic phase signal.

Components (*i*)	*A_i_*	*a_i_*_,1_	*a_i_*_,2_	*a_i_*_,3_
1	1.5	0.3	2×10^–3^	2×10^–5^
2	1.5	0.7	–5×10^–3^	–2×10^–5^

**Figure 4:**
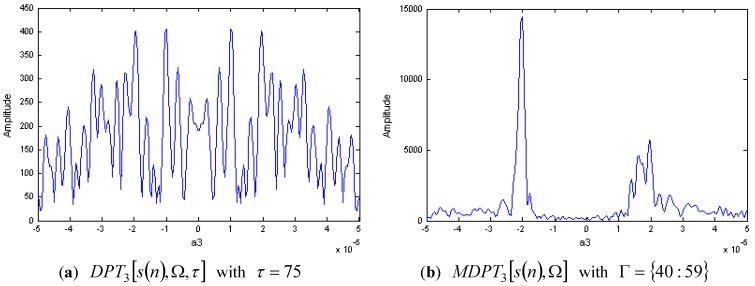
DPT and MDPT of a two-component CPS with same amplitudes.

The DPT of s(n) with M=3 and τ=75 is shown in Figure 4a. We can see that the individual signal component can not be detected correctly because of the cross-terms and the spurious peaks. This demonstrates that the traditional DPT algorithm is invalid for the signal components with the same amplitudes.

Figure 4b is the modified version of DPT with Γ={40:59}, it is obvious that the cross-terms and spurious peaks have been reduced greatly, and the individual signal component can be detected correctly. Then, the parameters of each component can be estimated by the peak of MDPT.

## 4. ISAR Imaging Algorithm Based on MDPT

In ISAR imaging of a maneuvering target, the echo signal in a range bin is characterized as a multi-component CPS after the motion compensation, and it is accurate enough to generate a focused ISAR image by the RID technique. In this section, the parameters of the multi-component CPS are estimated by the MDPT algorithm proposed in this paper, and the corresponding ISAR imaging algorithm is described in detail as follows:

Step 1: Motion compensation for the received signal. The envelope alignment can be implemented by the accumulated form of maximum correlation algorithm [19], and the phase adjustment can be implemented by the constant phase error elimination algorithm [19].

Step 2: Characterize the echo signal in a range bin as K components discrete CPS model, which is shown as Equation (18):

(18) $$s\left(n\right)=\sum_{i=1}^{K}A_{i}\exp\left[j\left(a_{i,1}n+a_{i,2}n^{2}+a_{i,3}n^{3}\right)\right],0\leq n\leq N-1$$

where N is the signal length, $a_{i,1},a_{i,2},a_{i,3}$ are the phase coefficients of ith component, $A_{i}$ is the amplitude of ith component, and the sampling rate is assumed to be one.

Step 3: Initialize i=1, and $s_{1}\left(n\right)=s\left(n\right)$.

Step 4: Apply $MDPT_{3}\left[s\left(n\right),\Omega \right]$ to the signal $s_{1}\left(n\right)$, and then we can obtain the estimated value of ${\hat{a}}_{i,3}$ by the peak detection technique.

Step 5: Based on the estimated value of third order coefficient, the cubic phase signal $s_{1}\left(n\right)$ can be dechirped into a LFM signal with $\exp\left(-j{\hat{a}}_{i,3}n^{3}\right)$.

Step 6: Estimate the second order coefficient $a_{i,2}$ by the peak of DPT for the LFM signal.

Step 7: The other parameters ${\hat{a}}_{i,1}$ and ${\hat{A}}_{i}$ can be obtained by the dechirp technique associated with the Fourier Transform [34].

Step 8: Subtract the estimated ith CPS from the original signal s(n) by the CLEAN technique [40].

Step 9: Set i=i+1, and repeat the above steps until i=K or the energy of residual signal is less than a threshold (5% of the original signal, for example).

## 5. ISAR Imaging Results

In this section, the ISAR imaging results for real data are provided to illustrate the effectiveness of the MDPT algorithm in this paper.

### 5.1. Real Aircraft Data

**Figure 5:**
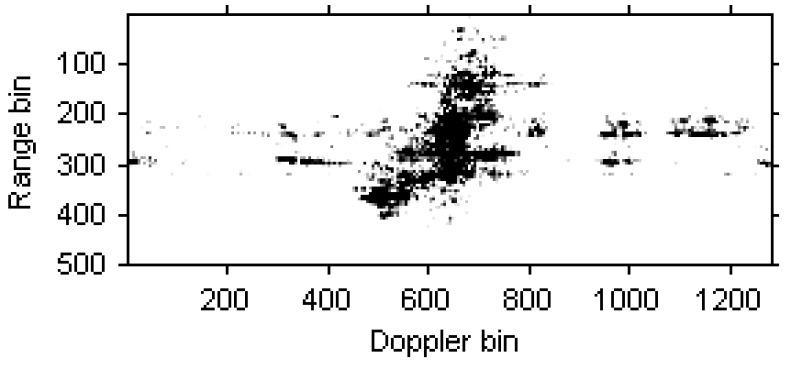
ISAR image based on the RD algorithm.

A set of real data of an aircraft target is selected here to demonstrate the effectiveness of the MDPT algorithm proposed in this paper. The center frequency is 5.52 GHz, the bandwidth is 400 MHz, and the pulse repeated frequency is 400 Hz. Figure 5 is the ISAR image based on the traditional RD algorithm. For the maneuverability of the target, the image is blurred severely.

**Figure 6:**
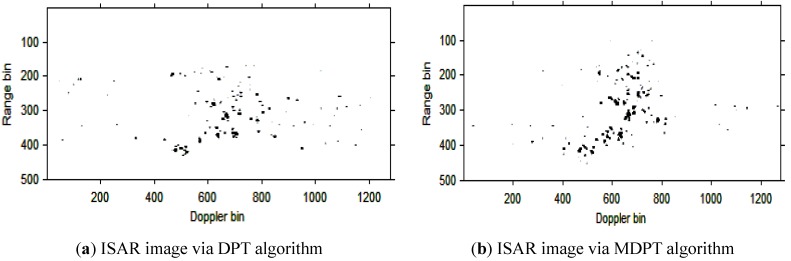
ISAR images at time *t* = 0.30 s based on DPT and MDPT algorithms.

The instantaneous ISAR images at time t=0.30s based on the cubic phase signal model are shown in Figure 6a,b. respectively. In Figure 6a, the DPT algorithm is used to estimate the parameters of each component; In Figure 6b, the MDPT algorithm with the sets of delay parameters selected as Γ={40:59} is used as an alternation to estimate the parameters of each component. Compared with Figure 6a and Figure 6b, we can see that the ISAR image quality in Figure 6b is better than that in Figure 6a, and the fake scatterers in Figure 6b have been reduced greatly.

**Figure 7:**
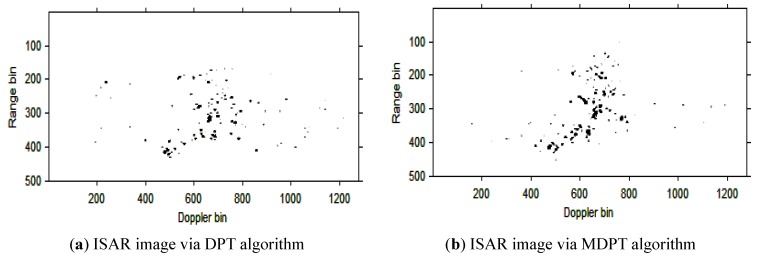
ISAR images at time *t* = 0.60 s based on DPT and MDPT algorithms.

**Figure 8:**
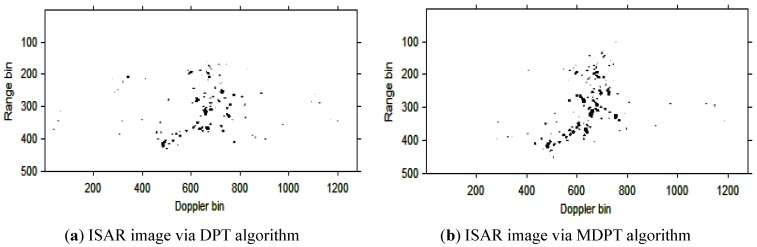
ISAR images at time *t* = 0.90 s based on DPT and MDPT algorithms.

**Figure 9:**
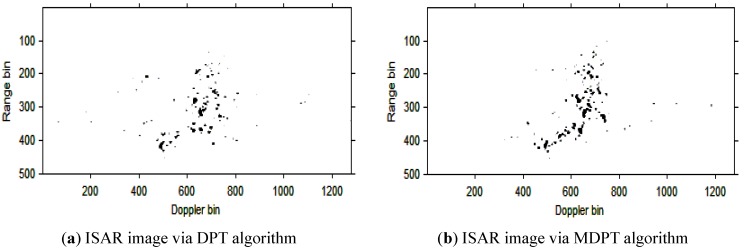
ISAR images at time *t* = 1.20 s based on DPT and MDPT algorithms.

**Figure 10:**
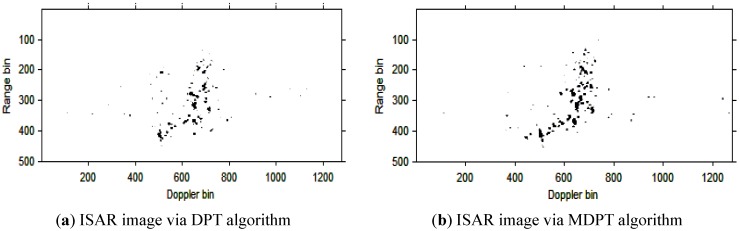
ISAR images at time *t* = 1.51 s based on DPT and MDPT algorithms.

**Figure 11:**
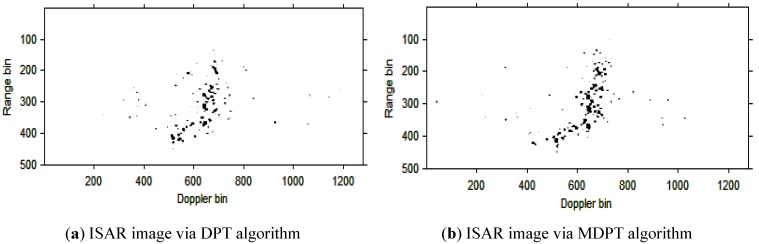
ISAR images at time *t* = 1.81 s based on DPT and MDPT algorithms.

Then, the instantaneous ISAR images at times *t* = 0.60 s, *t* = 0.90 s, *t* = 1.20 s, *t* = 1.51 s and *t* = 1.81 s based on DPT and MDPT algorithms are shown in Figure 7, Figure 8, Figure 9, Figure 10 and Figure 11, respectively.

From the ISAR images in Figure 7, Figure 8, Figure 9, Figure 10 and Figure 11, we can see that the MDPT algorithm is efficient to generate a well-focused ISAR images compared with the DPT algorithm.

### 5.2. Real Ship Data

**Figure 12:**
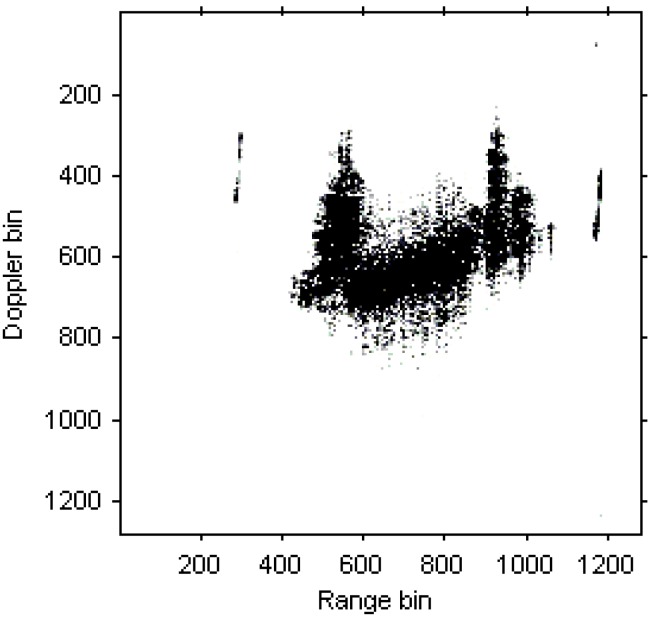
ISAR image based on RD algorithm.

A set of real data of ship target is used in this section, and the radar works at X band. The ISAR image based on RD algorithm is shown in Figure 12.

**Figure 13:**
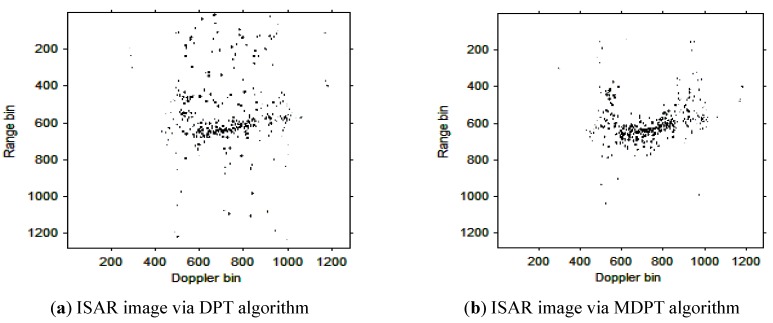
ISAR images at time *t* = 0.16 s based on DPT and MDPT algorithms.

**Figure 14:**
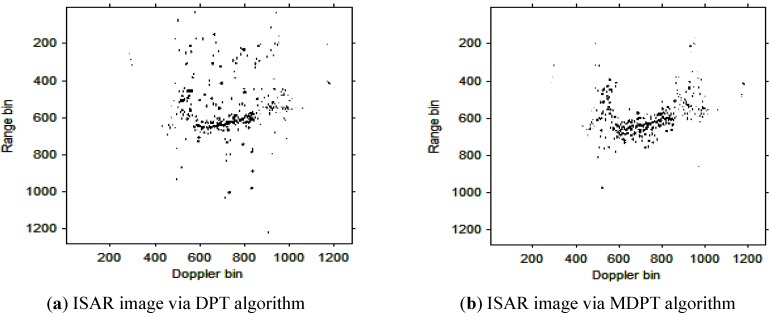
ISAR images at time *t* = 0.32 s based on DPT and MDPT algorithms.

**Figure 15:**
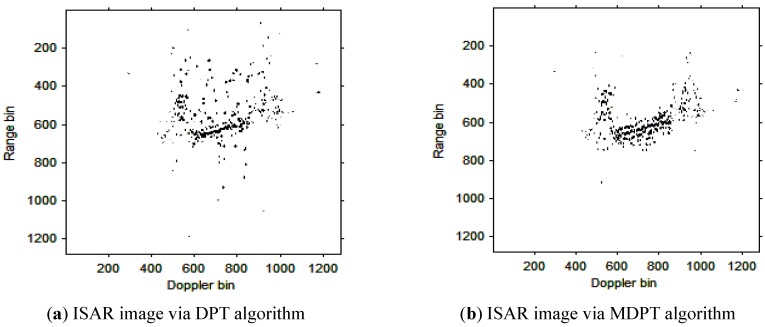
ISAR images at time *t* = 0.48 s based on DPT and MDPT algorithms.

**Figure 16:**
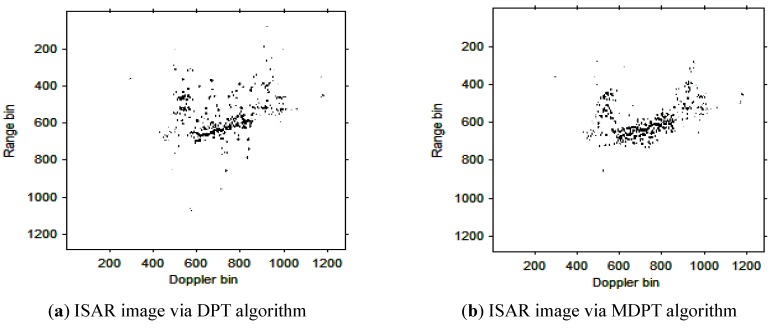
ISAR images at time *t* = 0.64 s based on DPT and MDPT algorithms.

**Figure 17:**
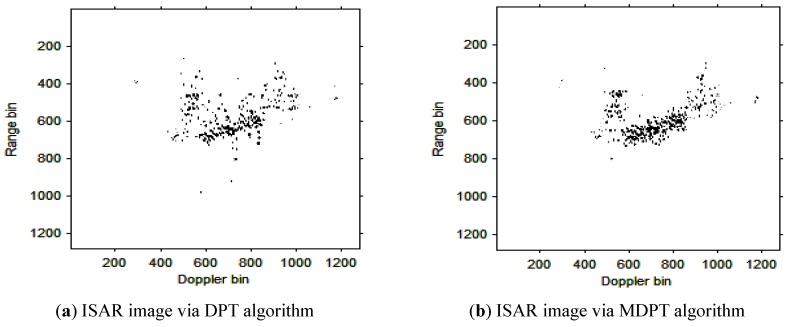
ISAR images at time *t* = 0.80 s based on DPT and MDPT algorithms.

**Figure 18:**
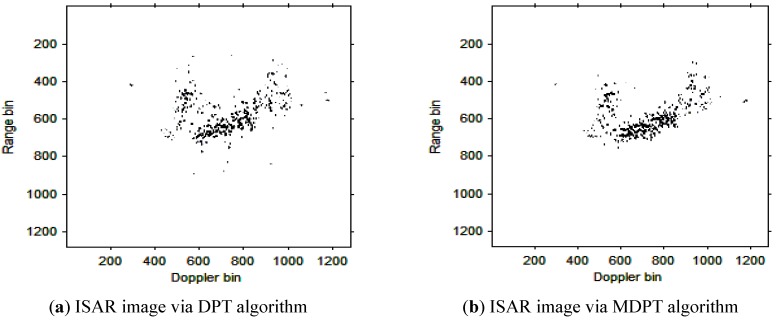
ISAR images at time *t* = 0.96 s based on DPT and MDPT algorithms.

The instantaneous ISAR images at time *t* = 0.16 s, *t* = 0.32 s, *t* = 0.48 s, *t* = 0.64 s, *t* = 0.80 s and *t* = 0.96 s based on DPT and MDPT algorithms are shown in Figure 13, Figure 14, Figure 15, Figure 16, Figure 17 and Figure 18, respectively.

From the ISAR images in Figure 13, Figure 14, Figure 15, Figure 16, Figure 17 and Figure 18, we can see that the MDPT algorithm is efficient to generate a well-focused ISAR images compared with the DPT algorithm.

Remark 2: The quality of ISAR images based on DPT and MDPT algorithms in this paper can be compared in two ways. The first is the fake scatterers in the ISAR images. As shown in Figure 6, Figure 7, Figure 8, Figure 9, Figure 10 and Figure 11 and Figure 13, Figure 14, Figure 15, Figure 16, Figure 17 and Figure 18, the influence of fake scatterers for the ISAR image based on MDPT algorithm is slighter than the ISAR image based on the DPT algorithm. Here, we can see that for the MDPT algorithm, there still exist fake scatterers in the corresponding ISAR images. An existing technique to remove the fake scatterers has been proposed in [34] by the statistical CLEAN technique. The aim of this paper is to demonstrate the advantage of MDPT algorithm in the ISAR imaging procedure compared with the traditional DPT algorithm. This has been implemented by the comparison of the fake scatterers in the ISAR images. The second way for the comparison of ISAR images based on DPT and MDPT algorithms is the focused performance. For the MDPT algorithm, the ISAR images are well-focused, and the target shape can be well recognized simultaneously, while for the DPT algorithm, the ISAR images are not well-focused, and this can be seen from Figure 6, Figure 7, Figure 8, Figure 9, Figure 10 and Figure 11 and Figure 13, Figure 14, Figure 15, Figure 16, Figure 17 and Figure 18.

Remark 3: The innovation of this paper can be summarized in two aspects. One is the presentation of the modified discrete polynomial transform (MDPT), which can be used in the parameters estimation of multi-component cubic phase signal. With the integration operation of MDPT algorithm, the individual signal components can be amplified and the cross-terms between different signal components can be reduced. Hence, the MDPT algorithm is more appropriate for the parameters estimation of multi-component cubic phase signal than the traditional DPT algorithm. The second is the novel ISAR imaging algorithm based on the MDPT method, and the ISAR images quality can be improved greatly for the maneuvering target. Some existing algorithms have been proposed for ISAR imaging of target with complex motion, but some of them suffer from the high order of nonlinearity or computational complexity. The MDPT algorithm in this paper can be implemented efficiently by one-dimensional maximizations with high accuracy, and thus it is superior to the traditional algorithms.

Remark 4: For [30] and [31], two different algorithms for the parameters estimation of multi-component polynomial phase signals were proposed. In [30], the product high order ambiguity function was proposed, and in [31], the multilinear function was proposed. The two algorithms are different from the MDPT algorithm in essence. Furthermore, the algorithms in [30] and [31] are just presented to estimate the parameters of polynomial phase signal without the application in ISAR imaging. The advantage of the MDPT algorithm in this paper is demonstrated by the comparison of ISAR images quality with the traditional DPT algorithm, and the conventional Doppler processing is not compared with the MDPT algorithm in this paper.

Remark 5: The cross range scaling should be implemented for the ISAR imaging procedure, and the authors have proposed some efficient algorithm on this topic, as shown in [41,42].

## 6. Conclusions

For ISAR imaging of a target with high maneuverability, the echo signal in a range bin can be considered as a multi-component CPS. In this paper, a novel algorithm for the parameters estimation of multi-component CPS by the MDPT method is proposed. This algorithm has better performance than the traditional DPT algorithm in the case of similar amplitudes of each component. Then, the corresponding ISAR imaging algorithm based on MDPT is presented. The ISAR imaging results of real data demonstrate the validity of the novel algorithm proposed in this paper.

## Nomenclature

O—Geometric center of the target

P—Random scatterer on the target

t—Azimuth time

V(t)—Composite velocity of a scatterer

K—Number of scatterers in a range bin

τ—Delay parameters

M—Order of the DPT

∗—Conjugate operator

A—Amplitude of a cubic phase signal

$a_{1}$—First order coefficient

$a_{2}$—Second order coefficient

$a_{3}$—Third order coefficient

N—Signal length

## References

[1] Thayaparan T., Lampropoudos G., Wong S.K., Riseborough E. (2003). Application of adaptive joint time-frequency algorithm for focusing distorted ISAR images from simulated and measured radar data. IEE Proc. Radar Sonar Navig..

[2] Li J.F., Ling H., Chen V.C. (2003). An algorithm to detect the presence of 3D target motion from ISAR data. Multidimension. Syst. Signal Process..

[3] Barbarossa S., Scaglione A. (1998). Autofocusing of SAR images based on the product high-order ambiguity function. IEEE Proc. Radar Sonar Navig..

[4] Liu Z.S., Wu R.B., Li J. (1999). Complex ISAR imaging of maneuvering targets via the Capon estimator. IEEE Trans. Signal Process..

[5] Trintinalia L.C., Ling H. (1997). Joint time-frequency ISAR using adaptive processing. IEEE Trans. Antennas Propag..

[6] Wang Y.X., Ling H., Chen V.C. (1998). ISAR motion compensation via adaptive joint time-frequency technique. IEEE Trans. Aerosp. Electron. Syst..

[7] Zheng J.B., Su T., Zhu W.T., Liu Q.H. (2014). ISAR imaging of targets with complex motions based on the Keystone time-chirp rate distribution. IEEE Geosci. Remote Sens. Lett..

[8] Zheng J.B., Su T., Zhang L., Zhu W.T., Liu Q.H. (2014). ISAR imaging of targets with complex motion based on the chirp rate-quadratic chirp rate distribution. IEEE Geosci. Remote Sens..

[9] Prickect M.J., Chen C.C. (1980). Principle of inverse synthetic aperture radar (ISAR) imaging. Electron. Aerosp. Syst. Conf..

[10] Martorella M., Giusti E., Demi L., Zhou Z., Berizzi F., Bates B. (2011). Target recongnition by means of polarimetric ISAR images. IEEE Trans. Aerosp. Electron. Syst..

[11] Martorella M., Pastina D., Berizzi F., Lombardo P. (2014). Spaceborne radar imaging of maritime moving targets with the Cosmo-SkyMed SAR system. IEEE J. Sel. Top. Appl. Earth Obs. Remote Sens..

[12] Qiu W., Martorella M., Zhou J.X., Zhao H.Z., Fu Q. (2015). Three-dimensional inverse synthetic aperture radar imaging based on compressive sensing. IEE Proc. Radar Sonar Navig..

[13] Liu L., Zhou F., Tao M.L., Zhao B., Zhang Z.J. (2015). Cross-range scaling method of inverse synthetic aperture radar image based on discrete polynomial-phase transform. IEE Proc. Radar Sonar Navig..

[14] Li G., Zhang H., Wang X.Q., Xia X.G. (2012). ISAR 2-D imaging of uniformly rotating targets via matching pursuit. IEEE Trans. Aerosp. Electron. Syst..

[15] Lanterman A.D., Munson D.C., Wu Y. (2003). Wide-angle radar imaging using time frequency distributions. IEE Proc. Radar Sonar Navig..

[16] Bai X., Tao R., Wang Z.J., Wang Y. (2014). ISAR imaging of a ship target based on parameter estimation of multicomponent quadratic frequency-modulated signals. IEEE Geosci. Remote Sens..

[17] Pastina D., Bucciarelli M., Lombardo P. (2010). Multistatic and MIMO distributed ISAR for enhanced cross-range resolution of rotating targets. IEEE Geosci. Remote Sens..

[18] Walker J.L. (1980). Range-Doppler imaging of rotating objects. IEEE Trans. Aerosp. Electron. Syst..

[19] Liu Y.T. (1999). Radar Imaging Technique.

[20] Wang J.F., Liu X.Z. (2007). Improved global range alignment for ISAR. IEEE Trans. Aerosp. Electron. Syst..

[21] Zhu D.Y., Wang L., Yu Y.S., Tao Q.N., Zhu Z.D. (2009). Robust ISAR range alignment via minimizing the entropy of the average range profile. IEEE Geosci. Remote Sens. Lett..

[22] Berizzi F., Mese E.D., Diani M., Martorella M. (2001). High-resolution ISAR imaging of maneuvering targets by means of the range instantaneous Doppler technique: Modeling and performance analysis. IEEE Trans. Image Process..

[23] Bao Z., Wang G.Y., Luo L. (1998). Inverse synthetic aperture radar imaging of maneuvering targets. Opt. Eng..

[24] Xia X.G., Wang G.Y., Chen V.C. (2002). Quantitative SNR analysis for ISAR imaging using joint time-frequency analysis-Short Time Fourier Transform. IEEE Trans. Aerosp. Electron. Syst..

[25] Wu Y., Munson D.C. Wide-angle ISAR passive imaging using smoothed pseudo Wigner-Ville distribution. Proceedings of the 2001 IEEE Radar Conference.

[26] Wang Y., Jiang Y.C. (2010). ISAR imaging of maneuvering target based on the L-Class of fourth order complex-Lag PWVD. IEEE Geosci. Remote Sens..

[27] Chen V.C., Qian S. (1998). Joint time-frequency transform for radar range-Doppler imaging. IEEE Trans. Aerosp. Electron. Syst..

[28] Wang G.Y., Bao Z., Sun X.B. (1996). Inverse synthetic aperture radar imaging of non-uniformly rotating targets. Opt. Eng..

[29] Wang Y., Jiang Y.C. (2012). Inverse synthetic aperture radar imaging of three-dimensional rotation target based on two-order match Fourier transform. IET Signal Process..

[30] Sun C.Y., Bao Z. (2000). Super-resolution algorithm for instantaneous ISAR imaging. Electron. Lett..

[31] Xing M.D., Bao Z. (2001). Translational motion compensation and instantaneous imaging of ISAR maneuvering target. Multispectr. Hyperspectr. Image Acquis. Process..

[32] Wang Y., Jiang Y.C. (2009). ISAR imaging of a ship target using product high order matched-phase transform. IEEE Geosci. Remote Sens. Lett..

[33] Wang Y., Kang J., Jiang Y.C. (2014). ISAR imaging of maneuvering target based on the local polynomial Wigner distribution and integrated high order ambiguity function for cubic phase signal model. IEEE J. Sel. Top. Appl. Earth Obs. Remote Sens..

[34] Wang Y., Jiang Y.C. (2011). Inverse synthetic aperture radar imaging of maneuvering target based on the product generalized cubic phase function. IEEE Geosci. Remote Sens. Lett..

[35] Peleg S., Friedlander B. (1995). The discrete polynomial-phase transform. IEEE Trans. Signal Process..

[36] Peleg S., Friedlander B. (1996). Multicomponent signal analysis using the polynomial-phase transform. IEEE Trans. Aerosp. Electron. Syst..

[37] Abatzoglou T. (1986). Fast maximum likelihood joint estimation of frequency and frequency rate. IEEE Trans. Aerosp. Electron. Syst..

[38] Barbarossa S., Scaglione A., Giannakis G.B. (1998). Product high-order ambiguity function for multicomponent polynomial-phase signal modeling. IEEE Trans. Signal Process..

[39] O’shea P., Wiltshire R.A. (2009). A new class of multilinear functions for polynomial phase signal analysis. IEEE Trans. Signal Process..

[40] Gough P.T. (1994). A fast spectral estimation algorithm based on the FFT. IEEE Trans. Signal Process..

[41] Wang Y., Jiang Y. (2008). A novel algorithm for estimating the rotation angle in ISAR imaging. IEEE Geosci. Remote Sens. Lett..

[42] Wang Y., Zhang R.B., Kang J. Rotational parameters estimation for ISAR imaging of maneuvering target. Proceedings of the 12th International Conference on Signal Processing.

